# Impacts of ontogenetic dietary shifts on the food-transmitted intestinal parasite communities of two lake salmonids

**DOI:** 10.1016/j.ijppaw.2020.06.002

**Published:** 2020-06-10

**Authors:** Sebastian Prati, Eirik Haugstvedt Henriksen, Rune Knudsen, Per-Arne Amundsen

**Affiliations:** Department of Arctic and Marine Biology, Faculty of Biosciences, Fisheries and Economics, UiT The Arctic University of Norway, Muninbakken 21, 9019, Tromsø, Norway

**Keywords:** Arctic charr, Brown trout, Trophic transmission, Subarctic, β-Diversity

## Abstract

Ontogenetic dietary shifts are common in fish and often impact trophically transmitted parasite communities. How parasite species composition and relative abundances change among size classes, and at what rate these changes occur, is rarely examined. Hosts with a broad trophic niche are potentially exposed to a large variety of parasite species. The degree of ontogenetic changes in parasite species composition versus changes in parasite abundance should suggestively differ between thropically generalist and specialist host species. In the present study, we explore ontogenetic dietary shifts and their impact on species composition and relative abundance of intestinal parasites in two sympatric salmonid fish species, Arctic charr (*Salvelinus alpinus*) and brown trout (*Salmo trutta*) caught in the littoral habitat of a subarctic lake. Our results highlight a close interplay between ontogenetic dietary niche shifts and alterations in the acquisition of trophically transmitted parasites, leading to host-specific differences in the component community of parasites. Ontogenetic changes in the intestinal parasite community related to dietary niche shifts were distinct but less pronounced in Arctic charr than in brown trout due to a broader and more consistent dietary niche of the former and an ontogenetic shift toward piscivory in the latter. At the component community level, changes in parasite assemblages of both host species were driven by a faster increase in the heterogeneity of parasite relative abundance than in the compositional heterogeneity, a pattern that partly may be related to a rather species-poor parasite community of this subarctic study system. Separating compositional heterogeneity from heterogeneity in relative parasite abundance is important to understand how size-dependent variability shapes parasite communities of host populations.

## Highlights

• Ontogenetic dietary niche shifts generated host-specific differences in the component community of parasites.
• Ontogenetic changes in the intestinal parasite community were less pronounced in Arctic charr than in brown trout.
• Heterogeneity of parasite relative abundance increased faster than the compositional heterogeneity.

## Graphical abstract

**Figure:**
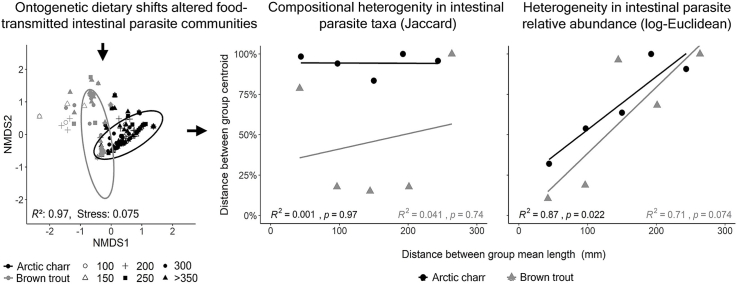

## 1 Introduction

While growing in body size, fish commonly experience profound physiological, morphological, and behavioral changes that often translate into ontogenetic dietary shifts (Sánchez-Hernández et al., 2019). Changes in diet are the result of body size affecting the feeding ability and size range of prey consumed by fish (Sánchez-Hernández et al., 2019; Werner and Gilliam, 1984). For instance, gape size or other mouth dimensions related to body size limit the ability to handle prey in fish that consume the whole prey (Sánchez-Hernández et al., 2012; Woodward and Warren, 2007). Additionally, individual growth and size increments are directly correlated with food consumption rates (Amundsen et al., 2007; Forseth et al., 1994; Sánchez-Hernández et al., 2019), and an increase in quantitative food intake may increase the exposure to trophically transmitted parasites (Poulin and Leung, 2011). Newly-hatched fish begin their life free of parasites, and minor differences in parasite exposure through ontogenetic shifts may thus generate substantial differences in parasite community structure among individual hosts as well as between different size classes (Timi et al., 2010; Timi and Lanfranchi, 2013). Indeed, changes in parasite communities throughout host ontogenesis are commonly reported in various fish species (Münster et al., 2015; Poulin, 2000; Poulin and Leung, 2011). As fish grow, we might therefore, expect changes in parasite diversity and abundance. However, to which extent ontogenetic changes in parasite communities among fish is driven by changes in taxa composition and/or by changes in their relative abundance remain poorly explored (see Timi et al., 2010; Timi and Lanfranchi, 2013).

In the present study, we explore ontogenetic dietary shifts and their impact on species composition and relative abundance of intestinal parasites in two sympatric living salmonid fish species; Arctic charr (*Salvelinus alpinus*) and brown trout (*Salmo trutta*). Arctic charr and brown trout like many other carnivorous fish, typically undergo ontogenetic dietary shifts from zooplankton to larger benthic macroinvertebrates and finally to piscivory (Amundsen et al., 2003; Jensen et al., 2008; Klemetsen et al., 2003; Sánchez-Hernández et al., 2012). In sympatric populations, the ontogenetic dietary transition from zooplanktivory to piscivory is generally more pronounced in brown trout than Arctic charr, as competition for resources frequently forces the latter to a more generalist feeding behavior (Eloranta et al., 2013; Knudsen et al., 2008; Sánchez-Hernández et al., 2017). Ontogenetic changes in diet are often reflected by changes in the intestinal parasite communities as observed in several fish species, including Arctic charr and brown trout (Brickle et al., 2006; Knudsen et al., 2008; Muñoz and Zamora, 2011; Münster et al., 2015).

As fish grow, one may expect an increase in parasite abundance and species richness as commonly observed (Poulin, 2000; Valtonen et al., 2010). However, in some species, smaller individuals harbor a more variable parasite community than larger ones (Timi et al., 2010). This could happen when small differences in the exposure of parasites at the younger stage generate substantial intraspecific differences, while the parasite community of larger and older fish becomes more homogeneous through repeated parasite exposure (Timi et al., 2011; Timi and Lanfranchi, 2013). Furthermore, core parasite species appearing in early-stage classes may archive higher prevalence and dominance in subsequent size classes, contributing to the homogenization of parasite communities in larger individuals (Pérez-Del Olmo et al., 2008).

Alternatively, we might expect a directional successional pattern in which certain food-transmitted parasites are going to be replaced by others, and/or that as colonization rates are generally higher than extinction rates repeated exposure and accumulation of long-lived parasites might result in an increased relative abundance of some taxa (Espínola-Novelo et al., 2020; Kuris et al., 1980; Timi et al., 2010). For instance, clear differences in parasite composition related to ontogenetic dietary shifts were observed in cod (*Gadus morhua*) and clingfish (*Sicyases sanguineus*) (Muñoz and Zamora, 2011; Münster et al., 2015).

However, how fast these changes occur and which processes are the driving force remain uncertain. Previous studies have demonstrated that β-diversity, i.e., the ratio between local and regional species diversity, is a suitable tool for analyzing the rate of changes in parasites community among fish of different size classes (Timi et al., 2010; Timi and Lanfranchi, 2013). For instance, Timi and Lanfranchi (2013) found that as fish grows, compositional heterogeneity of parasite assemblages in Argentinian congers (*Conger orbignianus*) increased faster than heterogeneity in relative abundances. Congers, like our focal species undergo ontogenetic dietary shifts and harbor various food transmitted gastrointestinal parasites (Anastasopoulou et al., 2013; Choi et al., 2008; Tanzola and Guagliardo, 2000). Accordingly, we have adopted the β-diversity concept to quantify and compare the rate of changes in parasite taxa composition and relative abundance among fish of different size classes.

The present study investigates whether changes in the intestinal parasite communities of Arctic charr and brown trout are related to ontogenetic shifts in diet and if these changes are due to variations in parasite taxa composition or changes in the relative abundance of acquired parasites. We firstly hypothesized that both salmonid species show distinct changes in the structure of their intestinal parasite communities related to their expected ontogenetic dietary shifts. Secondly, we hypothesized that variability in the structure of the intestinal parasite community in the generalist Arctic charr is driven by changes in the relative abundance of the parasite assemblages, while that of the specialist brown trout having more pronounced ontogenetic dietary shifts, to a larger extent is affected by changes in parasite taxa composition.

## 2 Materials and methods

### 2.1 Fish sampling

The fish sample consisted of 120 Arctic charr and 120 brown trout, collected between August 2017 and May 2018 using multi-meshed gillnets set overnight in the littoral habitat (<15 m depth) of Lake Takvatn. Takvatn is a dimictic oligotrophic subarctic lake, located in northern Norway. The only fish species present in the lake are Arctic charr, brown trout, and three-spined stickleback (*Gasterosteus aculeatus*) (see Amundsen et al. (2009) for further details about the lake). In the field, fork length in mm, weight, sex, and gonad maturation were recorded for all fish. Stomachs were opened, and the fullness degree was determined on a scale from 0 to 100% (Amundsen and Sánchez-Hernández, 2019). The stomach contents were preserved in 96% alcohol, and the intestines were frozen to preserve the content, allowing subsequent dietary and parasitological analyses in the laboratory (see Prati et al., 2020) for more detailed information on the sampling procedure).

### 2.2 Diet

Prey types were identified from the stomach and intestine of each fish. Only amphipods, insect larvae, zooplankton, and fish were considered for the present analysis, as they are the potential intermediate hosts of the identified intestinal parasites. Dietary information includes the whole gastrointestinal tract (stomach and intestine). As prey retrieved from the stomach and intestine showed different degrees of digestion and were not comparable with volumetric measures, dietary information is here expressed as the frequency of occurrence $f_{\left(\%\right)}=\frac{n\;of\;gastrointestinal\;tracts\;in\;wich\;a\;certain\;prey\;type\;occurred}{n\;of\;gastrointestinal\;tracts\;analyzed}\;*\;100$ (Amundsen and Sánchez-Hernández, 2019).

### 2.3 Parasites

Five trophically transmitted parasite taxa in their adult stages that use Arctic charr and brown trout as final hosts (i.e., *Crepidostomum* spp., *Cyathocephalus truncatus*, *Eubothrium salvelini*, *E. crassum,* and *Proteocephalus* sp.) and one taxon in its larval stage that use birds as definitive hosts (i.e., *Dibothriocephalus* spp.) were identified from the fish intestines (Table 1). The trematode genus *Crepidostomum* comprises at least four different species in Lake Takvatn, half of which has not yet been scientifically described (Soldánová et al., 2017). These are here grouped as *Crepidostomum* spp. as they are only distinguishable through molecular analysis (Soldánová et al., 2017).

**Table 1:** Parasites found in the intestine of Arctic charr and brown trout and their life cycle.

Parasite taxa	Stage	Intermediate hosts	Final hosts	Lifetime in the host
*Crepidostomum* spp. (trematode)	Adult	Amphipods/insect larvae	Arctic charr and brown trout	1 year (Thomas, 1958)
*Cyathocephalus truncatus* (cestode)	Adult	Amphipods	Arctic charr and brown trout	20–55 days (Okaka, 1984)
*Eubothrium salvelini* (cestode)	Adult	Copepods/fish	Arctic charr	1–2 year (Hanzelová et al., 2002)
*E. crassum* (cestode)	Adult	Copepods/fish	Brown trout	1–2 year (Hanzelová et al., 2002)
*Proteocephalus* sp. (cestode)	Adult	Copepods/fish	Arctic charr and brown trout	1 year (Scholz, 1999)
*Dibothriocephalus* spp. (cestode)	Larvae	Copepods/fish	Birds	Not known for unencysted plerocercoids

The cestode *Dibothriocephalus* spp. (formerly *Diphyllobothrium* (Waeschenbach et al., 2017)) consists of two species, *D. dendriticus* and *D. ditremus*. Both species have copepods as the first intermediate host, fish as the second intermediate host, and birds as the final host (Halvorsen, 1970; Vik, 1964). Small fish that are prey of larger piscivorous fish may also act as paratenic hosts (Henriksen et al., 2016; Kuhn et al., 2016a). In the fish host, the larval stage (plerocercoids) of *Dibothriocephalus* spp. is usually encysted on the stomach wall, muscle, or other parts of the viscera, but as the current study focused on the community of intestinal parasites, we only included unencysted larvae found in the intestine.

### 2.4 Statistical analysis

Descriptive and statistical analyses were performed with the open-source software Rstudio (version 1.1.423, Rstudio Inc.) based on R (version 3.5.1, R Core Team) and the R based software QPweb (version 1.0.14, Reiczigel et al. (2019)).

In both fish species, the sample was equally divided into six consecutive size classes consisting of 20 individuals and delimited by 50 mm size increment (100 - >350 mm range). A cut-off value of 100 mm was also tested and showed similar but less pronounced trends both in ontogenetic dietary shifts and in species composition and relative abundance of intestinal parasites. Among the different size classes of Arctic charr, the number of individuals sampled during the ice-free period (summer, autumn) and those sampled during the ice-covered period (early winter, late winter) did not differ significantly (Mann-Whitney, W = 23.5, P = 0.218). In brown trout, however, due to lower catches during the winter, the different size classes generally had a higher proportion of individuals sampled during the ice-free period (Mann-Whitney, W = 31.5, P = 0.037).

To assess differences in diet between different size classes of Arctic charr and brown trout, we used PERMANOVA (function “Adonis” in the R package “vegan”) on Jaccard distance matrices. To assess possible relationships between parasite taxa, diet, and host length a Canonical Correspondence Analysis (CCA) was used. The CCA included intensities of parasite taxa as the response variable and presence/absence of prey types and hosts body length as explanatory variables. ANOVA-like permutations (999 cycles, function “anova.cca” in the R package “vegan” (Oksanen et al., 2019)) were used to test which variables explained a significant portion of the variation in parasite abundances. To investigate differences in diet between Arctic charr and brown trout, the frequency of occurrence of prey types was tested with a binomial GLM accounting for seasonality, and then with ANOVA (type II).

To examine differences in parasite load between Arctic charr and brown trout, the mean number of species, abundance, intensity, prevalence, and mean intensity (Bush et al., 1997) were analyzed. The mean number of parasite taxa was preferred over parasite richness to address changes in the parasite communities throughout the ontogenesis of the two hosts because the parasite richness at the component community level ranged from four in the smallest size classes of fish to five in the medium and large size classes. Potential relationships between the mean number of parasite taxa and host length were analyzed with a quadratic regression. We calculated a 95% confidence interval using Sterne's method (Reiczigel, 2003) for the prevalence (the percentage of individuals infected by a particular parasite species in a host population), and the bias-corrected and accelerated bootstrap (Rózsa et al., 2000) for the mean intensity (the average number of a particular parasite species in the infected individuals of a host population). The prevalence was tested with a binomial generalized linear model (GLM), while the mean intensity, was tested with a negative binomial GLM. Both models were subsequently tested with ANOVA (type II). Since Arctic charr and brown trout were collected over several seasonal periods, all GLM models accounted for seasonality using presence/absence or intensity (number of parasite individuals of a particular species in a single infected host individual) as the response variable with length and season as the predictor. The host's sex was initially included as a covariate in the models, but did not influence parasite infections and was therefore excluded. To assess differences in parasite communities between different size classes of Arctic charr and brown trout, we used PERMANOVA (function “Adonis”) on Bray-Curtis abundances matrices, whereas nonmetric multidimensional scaling (NMDS) was used to illustrate the results. The Bray Curtis matrices were based on abundance data i.e., the number of a particular parasite species harbored by individuals of a host population. This approach was also used to analyze and illustrate the overall differences in parasite assemblages between the two hosts.

To analyze if parasite communities differ among size classes, β-diversity was calculated as the average distance from individuals to the group centroid for each size class using Jaccard index on presence/absence data of parasites and Euclidean distances on log-transformed abundance data, and the obtained values were then regressed against the average body length of each group (Anderson et al., 2011, 2006; Timi and Lanfranchi, 2013). For a comparable scale, the average distances to centroids obtained from the two indices were then expressed as a percentage of the maximum value for that index. Distances from centroids were obtained by placing observations into a Euclidean space represented by Principal Coordinate (PCO) using the function “betadisper” in the R package “vegan”. This procedure allows preserving original dissimilarities between observations obtained with non-Euclidean indices like Jaccard (Anderson et al., 2006; Gower, 1966; Legendre and Legendre, 2012). Jaccard index emphasizes compositional dissimilarity, while Euclidean distances emphasize changes in relative abundance (Anderson et al., 2006; Timi and Lanfranchi, 2013). To calculate and compare variation in the rate of change of β-diversity among size classes, pairwise distances obtained from the coordinate of group centroids in the Euclidean space were regressed against pairwise fish body length distances (Anderson et al., 2011). To archive a comparable scale in the y-axis, the distances between centroids obtained from Jaccard and Euclidean indices were expressed as a percentage of the maximum value for that index.

## 3 Results

### 3.1 Diet

**Fig. 1:**
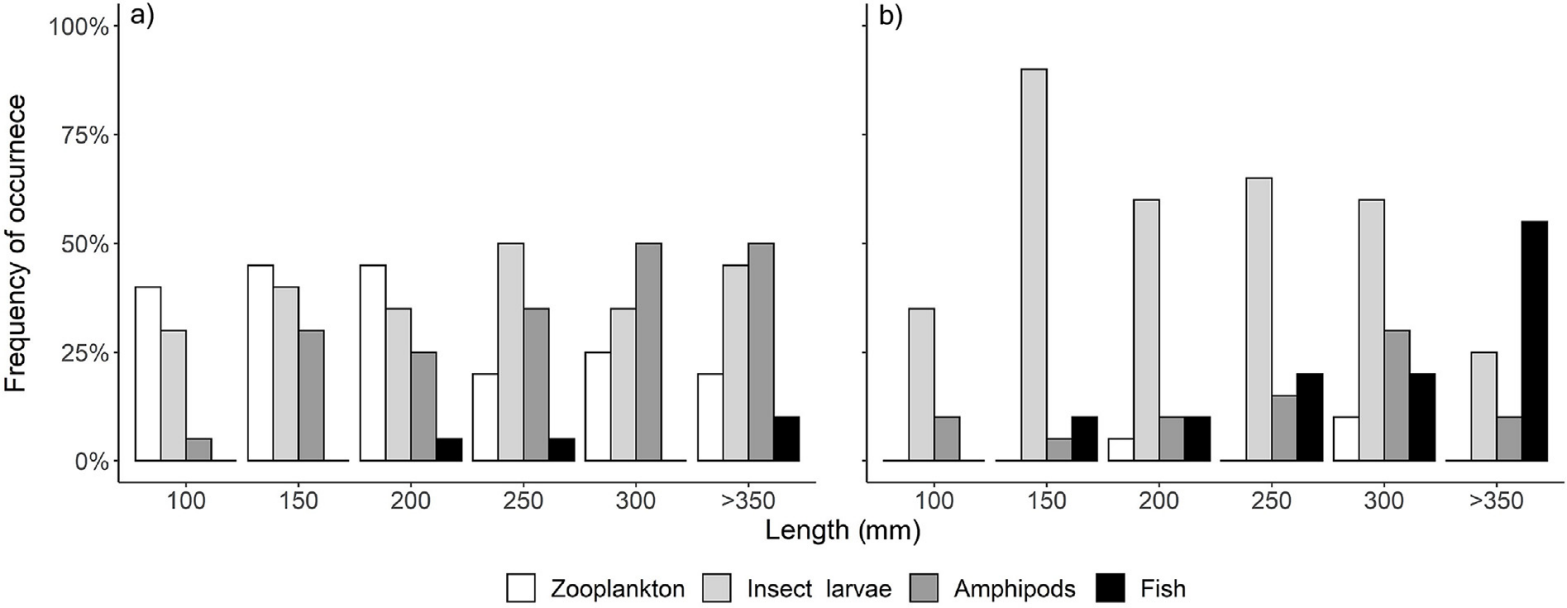
Frequency of occurrence of prey categories in the diet of a) Arctic charr and b) brown trout throughout their ontogenesis. Prey categories not related to intestinal parasite transmission are excluded.

The frequency of occurrence of prey types in the diet of Arctic charr was influenced both by season and body length, while that of brown trout was influenced only by its length (Table 2). The diet of both Arctic charr and brown trout differed significantly among size classes (PERMANOVA, F = 344.98, P < 0.001 and F = 320.25, P < 0.001), evidencing the occurrence of ontogenetic dietary shifts. For Arctic charr, insect larvae, zooplankton, and amphipods were the most common prey types (Fig. 1a). The occurrence of insect larvae remained reasonably stable in all size classes, while zooplankton was the most prevalent prey for Arctic charr under 250 mm, and amphipods were more common in fish over 250 mm. The least frequent prey was fish, which was absent in fish below 200 mm (Fig. 1a).

**Table 2:** Statistical results on variables associated with the frequency of occurrence of prey types in the diet of Arctic charr and brown trout (ANOVA form GLM binomial regression).

Prey	Variable	Arctic charr			Brown trout		
		χ^2^	Dƒ	P	χ^2^	Dƒ	P
Zooplankton	Length	9.91	1	0.002	0.30	1	0.583
	Season	41.96	3	<0.001	3.41	3	0.332
Insect larvae	Length	0.64	1	0.423	0.01	1	0.977
	Season	22.77	3	<0.001	5.50	3	0.139
Amphipods	Length	13.25	1	<0.001	0.21	1	0.647
	Season	11.49	3	0.009	5.86	3	0.119
Fish	Length	1.82	1	0.177	10.88	1	<0.001
	Season	2.69	3	0.441	3.21	3	0.361

In brown trout, insect larvae were the most prevalent prey type, except in the largest individuals (Fig. 1b). The importance of insect larvae decreased with increasing fish length, while the occurrence of fish prey steeply increased in fish larger than 350 mm. Amphipods were most commonly found in the 300–349 mm size group, but their overall contribution to the brown trout diet was modest. Zooplankton was absent in most size classes of brown trout (Fig. 1b).

### 3.2 The intestinal parasite communities of Arctic charr and brown trout

**Fig. 2:**
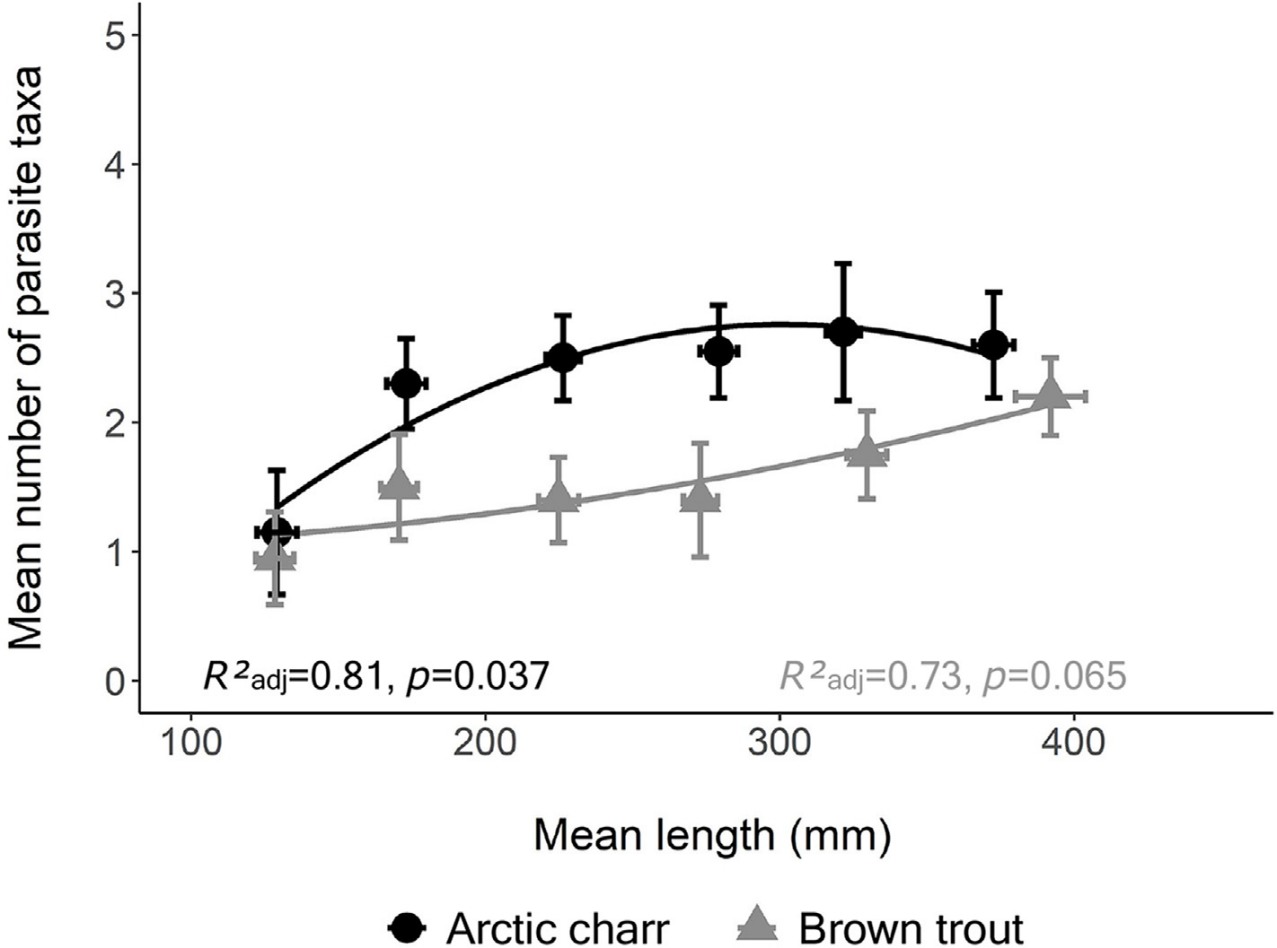
Correlation between the mean number of parasite taxa and mean length among the different size classes of Arctic charr and brown trout with a 95% confidence interval.

In both Arctic charr and brown trout, the mean number of parasite taxa increased throughout the ontogenesis, with Arctic charr harboring the highest parasite diversity in all size classes. The mean number of parasite taxa increased rapidly in the smaller size classes of Arctic charr before reaching an asymptote, whereas, for brown trout, it continued to increase linearly in the three largest size classes (Fig. 2).

**Fig. 3:**
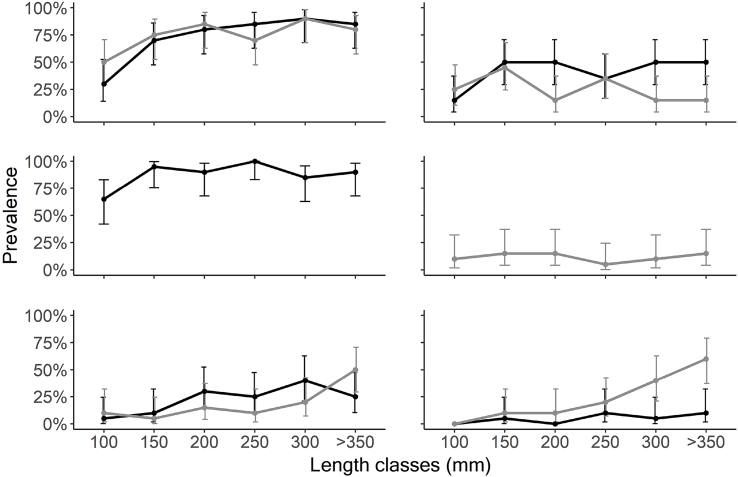
Prevalence of intestinal parasites in Arctic charr (black) and brown trout (grey) throughout their ontogenesis with 95% confidence intervals.

In Arctic charr, a total of five intestinal parasite taxa were recorded. Of these, *E. salvelini* and *Crepidostomum* spp. were the most prevalent within all size classes (Fig. 3). The prevalence of *Crepidostomum* spp., *E. salvelini,* and *C. truncatus* sharply increased in fish between 100 and 150 mm before stabilizing at relatively high levels. *Proteocephalus* sp., on the other hand, increased more gradually, reaching a peak in fish of 300 mm, followed by a decline in fish over 350 mm. *Dibothriocephalus* spp. in contrast, was absent in Arctic charr under 150 mm and between 200 and 250 mm, and the prevalence remained at low levels troughout the ontogeny (Fig. 3). Similarly to prevalence, the mean intensity of most of the intestinal parasites generally increased with increasing fish size (Supplementary material, Fig. S1).

Also in brown trout, a total of five intestinal parasite taxa were found. The most prevalent parasite was *Crepidostomum* spp., while *E. crassum* was the least common. The prevalence of *Proteocephalus* sp. and *Dibothriocephalus* spp. steeply increased in fish larger than 250 mm, whereas the latter was absent in fish under 150 mm (Fig. 3). Similar to what was observed in Arctic charr, the prevalence of *Crepidostomum* spp. had the most pronounced increase in brown trout below 150 mm and remained fairly stable at high values in fish over 200 mm. The prevalence of *C. truncatus*, in contrast, decreased with increasing host size, while that of *E. crassum* remained stable and at low levels (Fig. 3). The mean intensity of *E. crassum* had a marked peak in fish of 250–299 mm, while that of the other parasites remained relatively stable and at a low level (Supplementary material, Fig. S1).

**Fig. 4:**
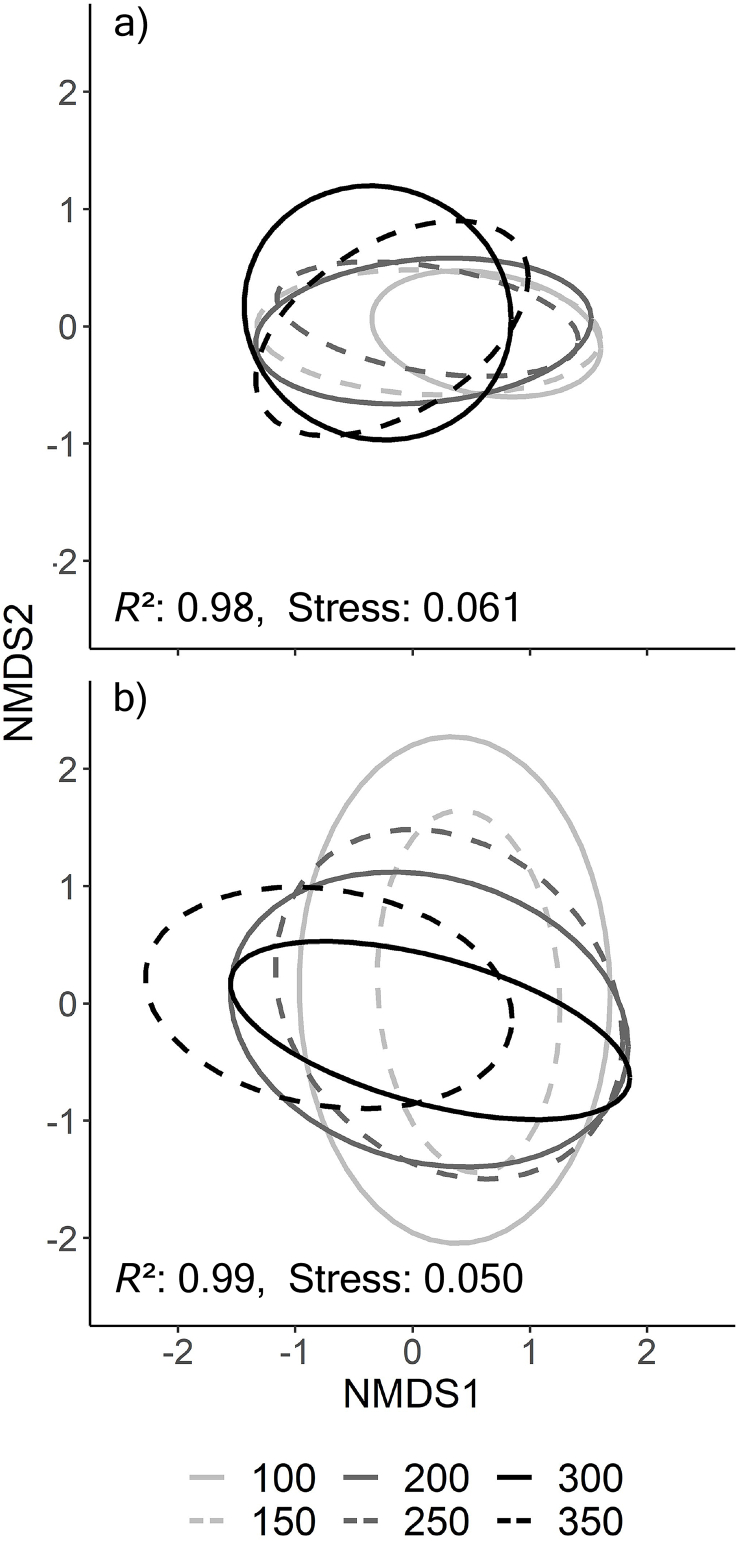
Nonmetric multidimensional scaling (NMDS) plot on Bray-Curtis distances of a) Arctic charr and b) brown trout showing dissimilarity in parasite community composition between different size classes including 95% confidence intervals ellipses. NMDS converged on a three-dimensional solution with an acceptable stress level.

**Fig. 5:**
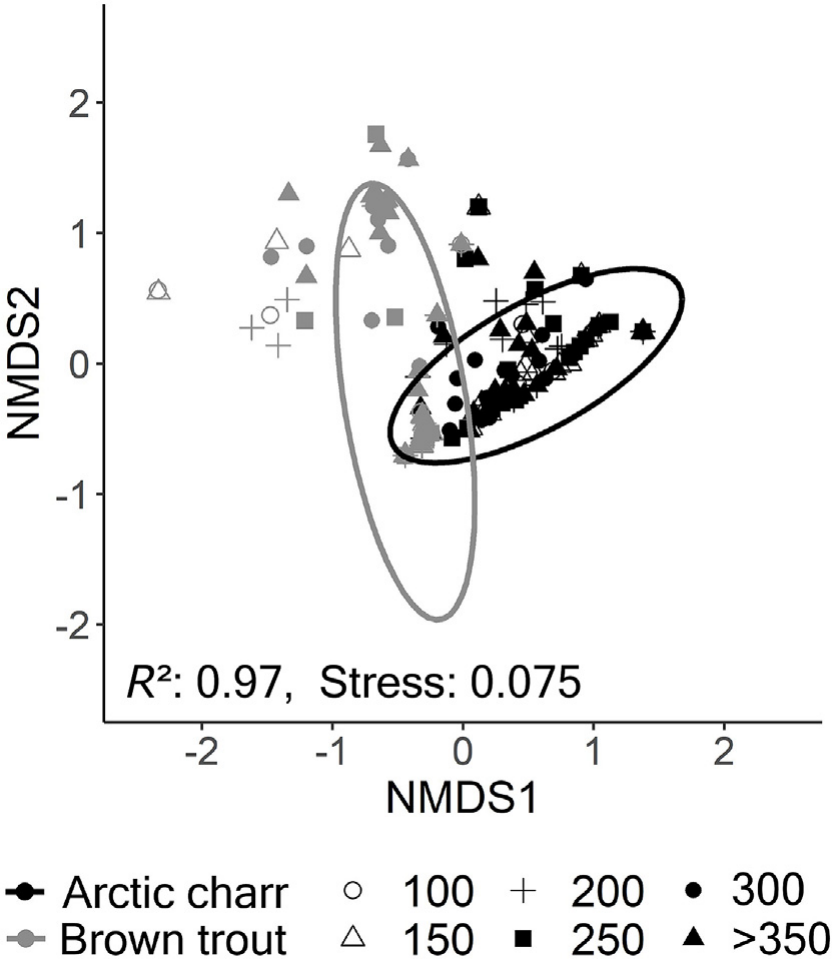
Differences in parasite community composition between Arctic charr and brown trout using nonmetric multidimensional scaling (NMDS) plot on Bray-Curtis distances, including 95% confidence interval ellipses. NMDS converged on a three-dimensional solution with an acceptable stress level.

Host length affected the intestinal parasite communities in both salmonids (Table 3a,b), except for the prevalence and intensity of *Dibothriocephalus* spp. in Arctic charr and *E. crassum* in brown trout. Dissimilarity in parasite abundances among size groups of both Arctic charr and brown trout were significant (PERMANOVA, F = 15.277, P < 0.001 and F = 6.104, P < 0.001) and mirrored by Bray-Curtis based NMDS plots (Fig. 4a and b). At the population level, the parasite communities of the two salmonids also significantly diverged from each other throughout host ontogeny (Fig. 5, PERMANOVA, F = 39.224, P < 0.001).

**Table 3:** Statistical result on variables associated with parasite intensity (ANOVA from GLM negative binomial regression) and prevalence (ANOVA form GLM binomial regression) in a) Arctic charr and b) in brown trout.

Parasite taxa	Variable	Prevalence			Intensity		
a) Arctic charr		χ^2^	Dƒ	P	χ^2^	Dƒ	P
*Crepidostomum* spp.	Length	19.11	1	<0.001	17.62	1	<0.001
	Season	5.05	3	0.168	4.48	3	0.215
*C. truncatus*	Length	4.57	1	0.032	19.29	1	<0.001
	Season	18.70	3	<0.001	6.09	3	0.107
*E. salvelini*	Length	4.21	1	0.040	30.34	1	<0.001
	Season	7.03	3	0.071	2.68	3	0.444
*Proteocephalus* sp.	Length	4.93	1	0.026	72.21	1	<0.001
	Season	7.44	3	0.059	10.69	3	0.014
*Dibothriocephalus* spp.	Length	1.87	1	0.171	1.99	1	0.158
	Season	1.32	3	0.723	2.18	3	0.536

b) Brown trout			Dƒ	P	χ^2^	Dƒ	P
*Crepidostomum* spp.	Length	6.61	1	0.010	13.41	1	<0.001
	Season	2.28	3	0.516	0.77	3	0.856
*C. truncatus*	Length	2.95	1	0.086	1.27	1	0.261
	Season	0.94	3	0.816	10.91	3	0.012
*E. crassum*	Length	0.10	1	0.748	0.12	1	0.734
	Season	17.26	3	<0.001	18.80	3	<0.001
*Proteocephalus* sp.	Length	14.02	1	<0.001	12.96	1	<0.001
	Season	3.34	3	0.343	4.14	3	0.247
*Dibothriocephalus* spp.	Length	51.84	1	<0.001	28.58	1	<0.001
	Season	10.03	3	0.018	9.25	3	0.026

**Fig. 6:**
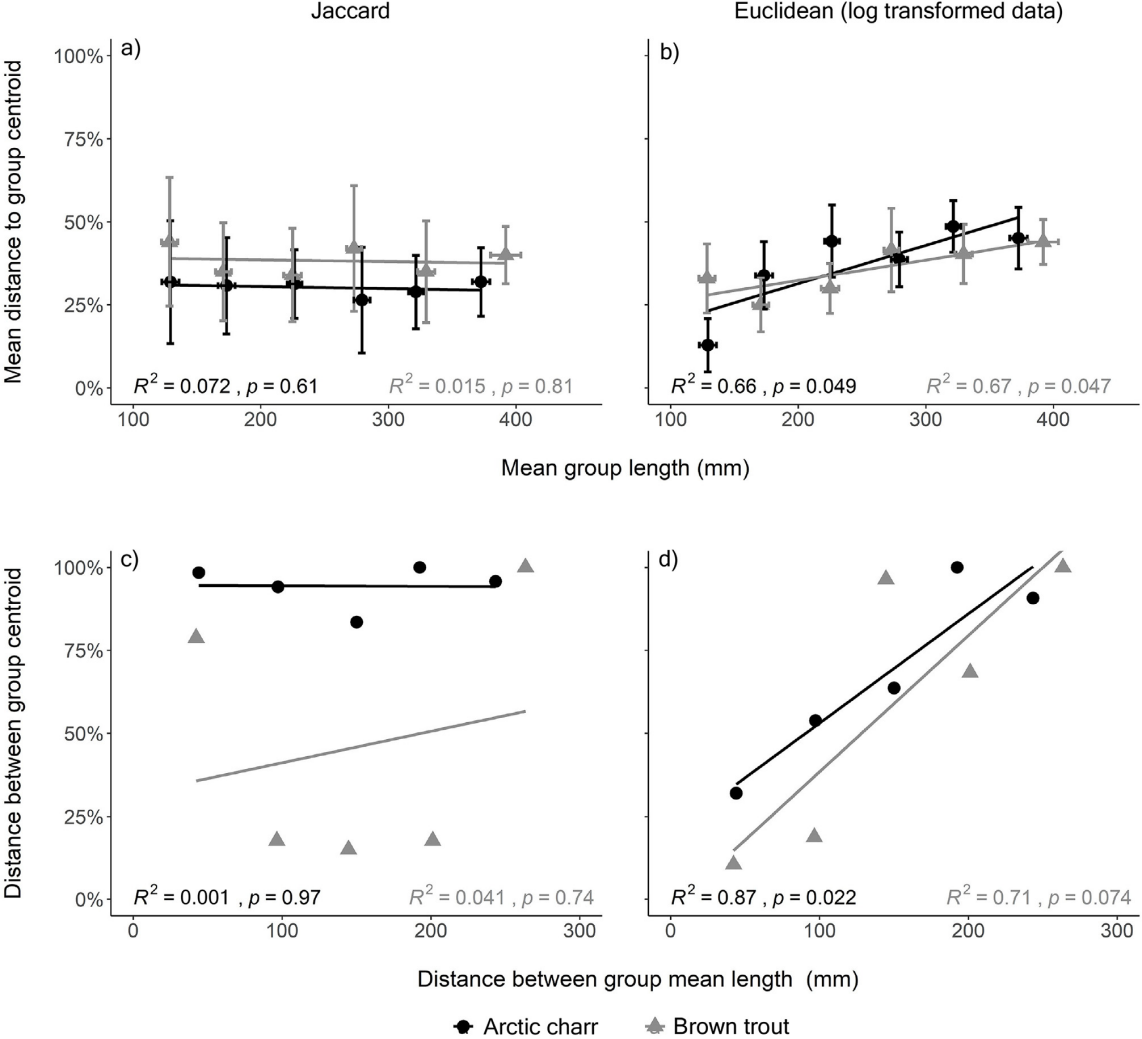
a) compositional heterogeneity and b) relative abundance of parasite assemblages measured as the average distance of individuals to group centroids as a function of the mean group length of the six different size classes. Rate of change of c) compositional heterogeneity and d) relative abundance of parasite assemblages measured as pairwise mean differences between group centroids and the average distances in groups mean length. Mean distances to group centroids and distances between group centroids are expressed as a percentage of the maximum value for the dissimilarity index used.

Variations in β-diversity among size classes revealed different patterns in the average distance to centroids depending on the dissimilarity measure used. Jaccard index, which emphasizes compositional differences in intestinal parasite communities, showed that the compositional variability of the parasite assemblages remained relatively stable among different size classes in both Arctic charr and brown trout (Fig. 6a). This indicates that the heterogeneity in parasite composition does not increase nor decrease significantly with increasing host size. In contrast, the Euclidean distances of log-transformed data, which emphasizes differences in relative abundances, indicated that there was a significant increase in the heterogeneity of parasite relative abundances with increasing body length for both host species (Fig. 6b).

The rate of change in β-diversity among different size classes remained relatively constant in Arctic charr, while that of brown trout increased toward the largest size class (Fig. 6c). Parasite taxa composition in brown trout was more dissimilar for 200–249 mm and >350 mm fish compared to the smallest size class. However, correlations between mean centroid distances and mean length distances were not significant. Increased dissimilarity in the relative abundances of parasites with increasing length distances was evident in both salmonids (Fig. 6d). In both Arctic charr and brown trout, the steeper regression slopes of the log-transformed Euclidean data compared to those obtained from the Jaccard index (2,63 versus −0,26 for Arctic charr and 1,72 versus 0,43 for brown trout) indicate that the heterogeneity in relative parasite abundance increased over ten times faster than the compositional heterogeneity.

### 3.3 Associations between parasites and diet

**Fig. 7:**
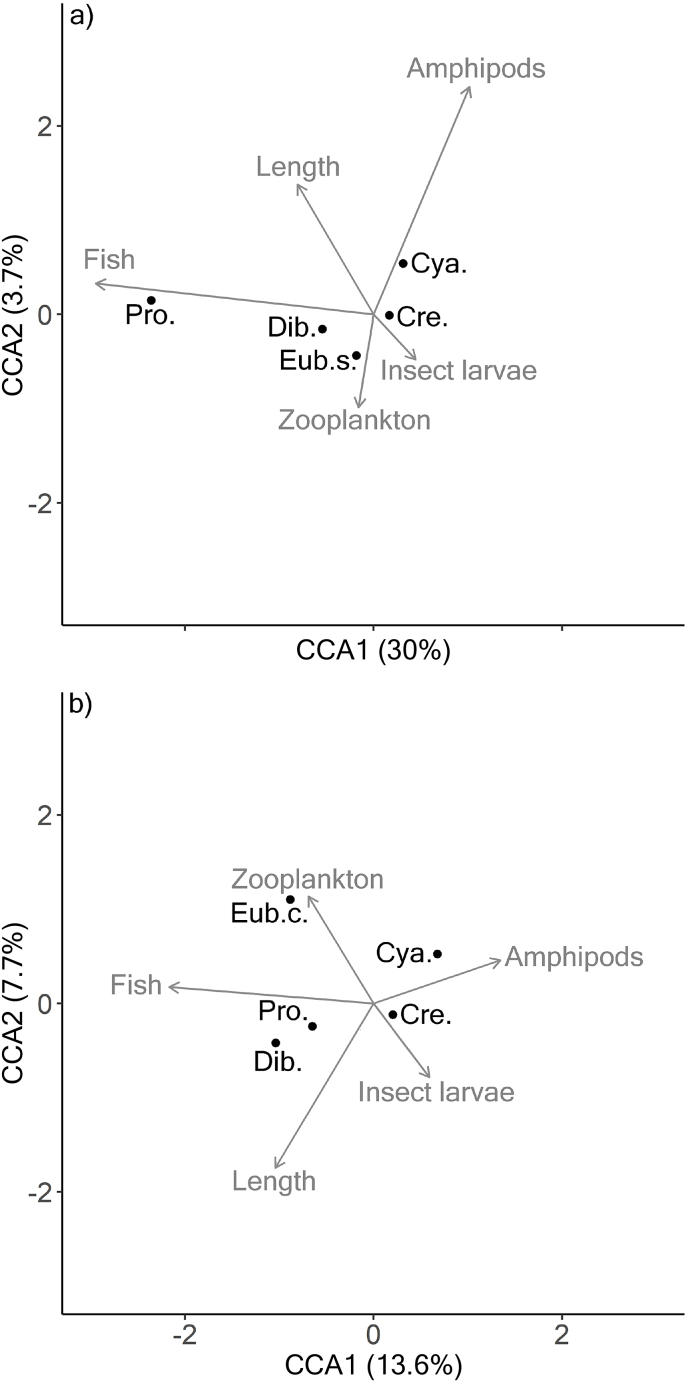
Canonical correspondence analysis (CCA) performed on parasite abundances as a function of presence-absence of prey types and fish length in a) Arctic charr and b) brown trout (Cre. = *Crepidostomum* spp., Cya. = *Cyatocephalus truncatus*, Eub.s. = *Eubothrium salvelini*, Eub.c. = *Eubothrium crassum*, Pro. = *Proteocephalus* sp., Dib. = *Dibothriocephalus* spp.).

In Arctic charr, variation in parasite abundances was significantly associated with the frequency of occurrence of amphipods and fish in the diet (CCA; permutation test, all P < 0.05). Together the first two dimensions of the CCA accounted for 33.7% of the total variation (Fig. 7a). Dimension 1 was mostly correlated with the explanatory variable fish prey and accounted for 30% of the total variation in the parasite abundance data, while dimension 2 was mostly correlated with amphipod predation and accounted for 3.7% of the total variation (Fig. 7a). In brown trout, variation in parasite abundances was significantly associated with host length and the frequency of occurrence of fish prey, amphipods and zooplankton in the diet (CCA; permutation test, all P < 0.05). The first two dimensions explained 21.3% of the total variation (Fig. 7b). Dimension 1, which accounted for 13.6% of the total variation, was mostly correlated with fish prey and amphipods in the diet, while dimension 2 was mainly driven by host length and predation on zooplankton and explained 7.7% of the total variation (Fig. 7b).

## 4 Discussion

Fish size strongly influenced both the diet and the structure of the intestinal parasite communities of Arctic charr and brown trout. Ontogenetic changes in parasite communities may result from changes in either species composition or relative abundance, or from both (Timi and Lanfranchi, 2013). Parasite taxa composition remained relatively stable throughout the ontogeny of both fish species, and the changes in the parasite communities were driven by variation in the relative abundance of different parasite taxa. However, our study addressed the only two fish species present at the highest trophic level within our lake system, which is characterized by a species-poor fauna and a relatively simple food-web (Amundsen et al., 2009, 2019). Hence our findings might differ from those of more complex freshwater systems.

As firstly hypothesized, the ontogenetic changes in the intestinal parasite community related to ontogenetic diet shifts were less pronounced in Arctic charr than in brown trout. Arctic charr displayed a more opportunistic diet compared to brown trout, with individuals of all size classes feeding on a larger variety of prey. The broader trophic niche of Arctic charr likely enhances the exposure to a wider variety of food-transmitted parasites resulting in higher parasite diversity, as has been shown for other fish species (Holmes, 1990; Kennedy et al., 1986; Locke et al., 2014). Brown trout typically reside all year round in the littoral habitat, whereas Arctic charr might exhibit ontogenetic differences in habitat use during the ice-free period, with large sized charr predominantly utilizing the littoral and pelagic habitat and small charr utilizing the profundal habitat (Klemetsen et al., 1989, 2002). However, during the ice-covered period, all size classes of Arctic charr typically converge into the littoral habitat (Amundsen and Knudsen, 2009; Knudsen et al., 2008). In the present study, all fish were caught in the littoral zone, and therefore the different size classes of Arctic charr are assumed to be equally able to utilize littoral resources.

As Arctic charr feeds on zooplankton, insect larvae, and amphipods throughout the entire ontogeny, the parasites transmitted by these intermediate hosts continue to establish, resulting in a more stable parasite community throughout the lifespan of Arctic charr. Accordingly, after an initial increase, the mean number of parasite taxa reached a plateau in the larger size classes. Moreover, Arctic charr mainly hosted parasites that can survive for about one year (or more) in the host intestine, including *Crepidostomum* spp., *E. salvelini*, and *Proteocephalus* sp. (Hanzelová et al., 2002; Hernandez and Muzzall, 1998; Scholz, 1999; Thomas, 1958), which contribute to stabilize the parasite diversity over time.

In brown trout, the ontogenetic variation in the parasite community was more pronounced, apparently reflecting the gradual shifts in the diet from benthivory to piscivory, as found in earlier studies (Jensen et al., 2012; Sánchez-Hernández et al., 2012). Accordingly, the mean number of parasite taxa gradually increased toward the largest size classes. The ontogenetic shift from benthivory to piscivory corresponded with decreased prevalence of parasites transmitted by benthic macroinvertebrate (i.e., the amphipod-transmitted *C. truncatus*) and an increase of those that can potentially be transmitted by fish prey as a paratenic host (i.e., *Proteocephalus* sp. and *Dibothriocephalus* spp.). Although the brown trout sample was skewed toward the ice-free period and piscivory might be more common during winter (Prati et al., 2020), there were no significant dietary differences among individuals captured in the different seasons (Table 2). Larger brown trout individuals frequently consume tree-spined sticklebacks, a key species with extensive linkages to the parasite community in the food web of Lake Takvatn (Amundsen et al., 2009, 2013). Three-spined stickleback may act as an intermediate host for several parasite species, including *Proteocephalus* spp., *Dibothriocephalus* spp. and *E. crassum* (Henriksen et al., 2016; Kristmundsson and Richter, 2009; Kuhn et al., 2016a). Hence, the transmission of *Proteocephalus* spp. and *Dibothriocephalus* spp. to brown trout appears to be size-dependent. Accordingly, brown trout gape size and handling ability increase proportionally with increased body size, allowing, the consumption of fish prey once reached a suitable size (Jensen et al., 2008; Johnson et al., 2006). A switch toward piscivory is a common choice for a predator like brown trout as it might result in enhanced growth rate and longevity (Hughes et al., 2019). Size-dependent piscivory has also been observed in many other freshwater fish species, including yellow perch (*Perca flavescens)*, burbot (*Lota lota)*, and pike (*Esox Lucius)* (Kahilainen and Lehtonen, 2003; Nilsson and Brönmark, 2000; Truemper and Lauer, 2005) and may shape parasite communities through enhanced transmission of particular parasite species. For instance, European eel (*Anguilla anguilla*) individuals with broader heads (i.e., increased gape size) tend to be piscivorous and have increased probability of infection by the nematode parasite *Anguillicoides crassus* due to its transmission via paratenic fish hosts (Pegg et al., 2015).

Our second hypothesis stated that changes in the intestinal parasite community of Arctic charr are driven by changes in the relative abundance of the parasite assemblage, while that of brown trout by compositional changes in parasite taxa. Our results, however, revealed that for both salmonids, heterogeneity in the relative abundance of parasite assemblages increased faster than the compositional heterogeneity. At smaller sizes, Arctic charr has a broader dietary spectrum than brown trout, and accordingly, larger chances of being exposed to a broader range of parasites (Locke et al., 2014). Even so, the various Arctic charr individuals do not consume the same types and amounts of prey, and consequently, they acquire different parasite assemblages, both qualitatively and quantitatively. Individual Arctic charr often specializes in specific prey types (like, e.g., amphipods or zooplankton) over a long period of time and thus acquire different parasites (Amundsen, 1995; Curtis et al., 1995; Knudsen et al., 2010, 2004). As shown by Dick et al. (2009) in a study on Shorthorn sculpin (*Myoxocephalus scorpius*), even if the diet range might be limited, individual fish feeding extensively on a few potential intermediate hosts should harbor heavier parasite loads compared to those feeding also on other prey taxa. However, the high degree of trophic specialization between Arctic charr individuals, whose dietary niches are sub-sets of the overall population trophic niche, continuously exposes the population to the same set of parasites (Knudsen et al., 2010). Consequently, at the component community level, variations in compositional heterogeneity of parasite assemblages with increasing fish size are less likely to arise, while increased heterogeneity in parasite relative abundance might result from individual feeding specialization and accumulation of long-lived parasites. The relative abundance of the different parasite taxa might also be influenced by competitive interactions among co-occurring intestinal helminth species, but previous studies in Lake Takvatn have shown that such interactions seem not to be important structuring force for the present parasites (Kuhn et al., 2016b).

Brown trout, despite undergoing more pronounced ontogenetic dietary shifts compared to Arctic charr, is restricted to a smaller number of prey types, as also seen in other lakes in which the two salmonids co-occur (Björnsson, 2001; Eloranta et al., 2013). Hence, the poorer parasite assemblage of brown trout is likely the result of predation constraints for a wider array of potential intermediate hosts. Moreover, a gradual transition in the ratios of invertebrates versus fish eaten and the relatively high presence of long-lived parasites might further mitigate changes in compositional heterogeneity of the parasite assemblages at the component community level. Piscivory is often linked to higher parasite diversity (Chen et al., 2008; Valtonen et al., 2010), and stronger compositional changes can therefore be expected in the parasite communities of piscivorous fish. This was not the case with brown trout in the present study, probably because there is only a limited number of parasite taxa that utilize both copepods and fish prey as intermediate hosts in our study system. The importance of piscivory in brown trout increased with increasing fish size, becoming the dominant form of feeding for the largest fish. Similar to what has been observed in other piscivorous fish (Bush et al., 1993; Marcogliese, 2002; Poulin and Valtonen, 2001), brown trout are exposed to large packages of several helminths taxa trough predation on infected fish prey. Hence, parasites acquired through piscivory contributed to the increased heterogeneity in the relative parasite abundance observed with increased brown trout size.

Overall, the use of multivariate dispersion as a measure of β-diversity was applicable in our system, but our results are in contrast with those of Timi and Lanfranchi (2013) who found that as fish grows, compositional heterogeneity of parasite assemblages in Argentinian congers (*Conger orbignianus*) increased faster than heterogeneity in relative abundances. The discrepancies between our study and that of Timi and Lanfranchi (2013) may have been influenced by the different complexity of the two systems studied. In contrast to temperate freshwater and marine systems, oligotrophic subarctic lakes are relatively simple systems with low biological diversity (Cañedo-Argüelles et al., 2017; Thomas et al., 2016), and consequently the number of parasite taxa that potentially may infect the hosts is limited. Hence, heterogeneity in parasite taxa composition is less likely to rise significantly, explaining the discrepancies in the observed pattern.

In conclusion, our study demonstrates a close interplay between ontogenetic dietary niche shifts and alterations in the acquisition of trophically transmitted parasites, leading to host-specific differences in the component community of parasites. The ontogenetic changes in the intestinal parasite community were distinct but less pronounced in Arctic charr than in brown trout due to a broader and more consistent dietary niche of the former. At the component community level, changes in parasite assemblages of both host species were driven by heterogeneity in relative abundance rather than compositional heterogeneity.

## Author contributions

S.P., E.H.H., R.K. and P.-A.A. conceived the idea and designed the methodology; S.P., E.H.H. and P.-A.A. conducted fieldwork; S.P. analyzed the data; S.P. led the writing on the manuscript with additional contributions from E.H.H., R.K. and P.-A.A. All authors contributed critically to the drafts and gave final approval for publication.

## Ethical approval

All applicable institutional and/or national guidelines for the care and use of animals were followed.

## Data accessibility

Prati, S., Henriksen, E.H., Knudsen, R., Amundsen, P.-A., 2020. Impact of ontogenetic dietary shifts on the food-transmitted intestinal parasite communities of two lake salmonids. Mendeley Data, https://doi.org/10.17632/xrsd8xt8yb.1.

## Declaration of competing interest

None.
