# Supplementary material for: Impacts of ontogenetic dietary shifts on the food-transmitted intestinal parasite communities of two lake salmonids
Source: Int J Parasitol Parasites Wildl. 2020 Jun 10;12:155–64. doi: 10.1016/j.ijppaw.2020.06.002 (PMC7300134; doi:10.1016/j.ijppaw.2020.06.002)
Supplement: Multimedia component 1 [file mmc1.docx]

**Supplementary material**


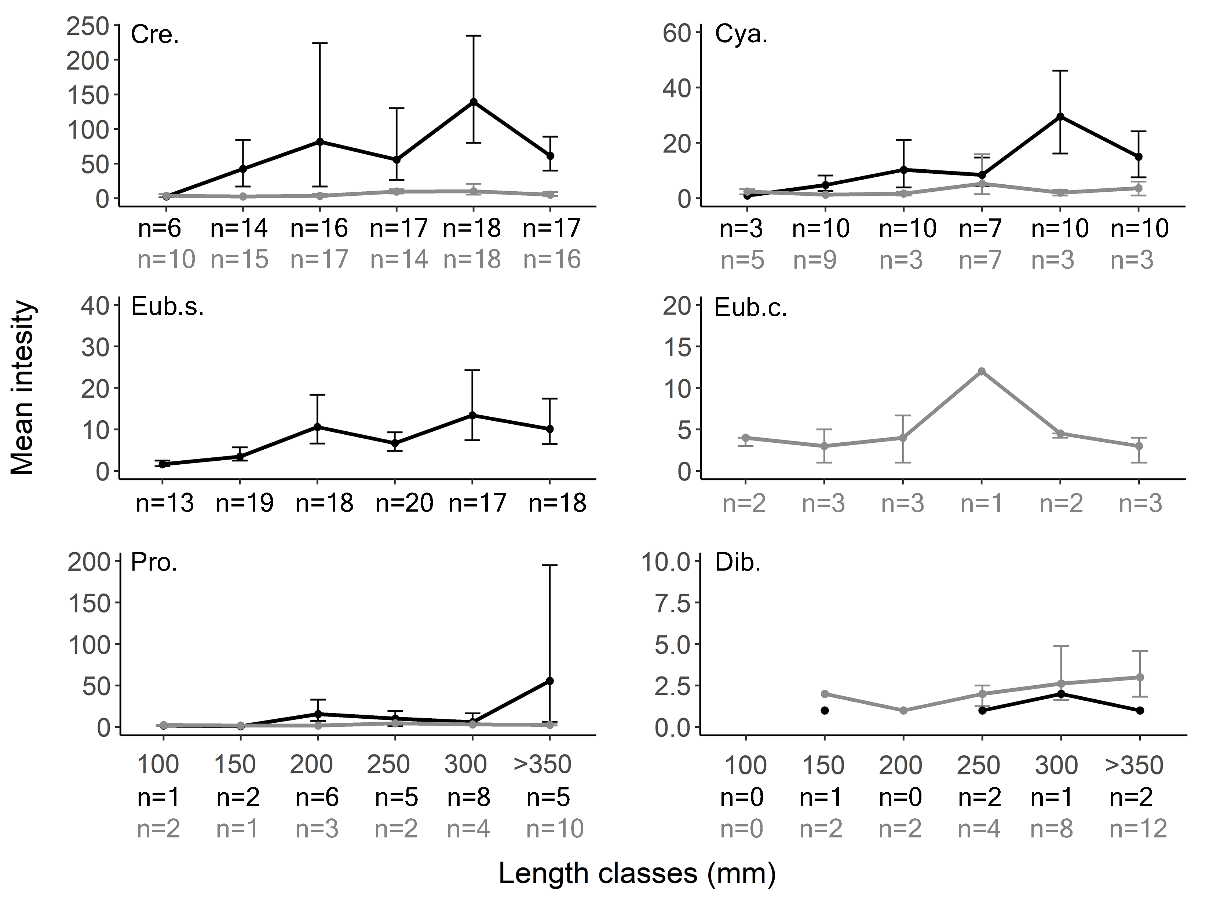


**Fig. S1** Mean intensity of intestinal parasites in Arctic charr (black) and brown trout (grey) throughout their ontogenesis with 95% confidence intervals.
